# The role of antioxidant response and nonphotochemical quenching of chlorophyll fluorescence in long-term adaptation to Cu-induced stress in *Chlamydomonas reinhardtii*

**DOI:** 10.1007/s11356-023-27175-y

**Published:** 2023-04-27

**Authors:** Bartosz Pluciński, Beatrycze Nowicka, Andrzej Waloszek, Joanna Rutkowska, Kazimierz Strzałka

**Affiliations:** 1 Department of Plant Physiology and Biochemistry, Faculty of Biochemistry, Biophysics and Biotechnology, Jagiellonian University, Gronostajowa 7, 30-387 Kraków, Poland; 2 Institute of Environmental Sciences, Faculty of Biology, Jagiellonian University, Gronostajowa 7, 30-387 Kraków, Poland; 3 Malopolska Centre of Biotechnology, Jagiellonian University in Kraków, Gronostajowa 7a, 30-387 Kraków, Poland

**Keywords:** *Chlamydomonas reinhardtii*, Chlorophyll fluorescence, Evolution, Heavy metal-induced stress, Peroxidase, Plastoquinone, Tocopherol

## Abstract

Copper is an essential micronutrient, but at supraoptimal concentrations it is also highly toxic, inducing oxidative stress and disrupting photosynthesis. The aim of the present study was to analyze selected protective mechanisms in strains of *Chlamydomonas reinhardtii* adapted and not adapted for growth in the presence of elevated copper concentrations. Two algal lines (tolerant and non-tolerant to high Cu^2+^ concentrations) were used in experiments to study photosynthetic pigment content, peroxidase activity, and non-photochemical quenching. The content of prenyllipids was studied in four different algal lines (two of the same as above and two new ones). The copper-adapted strains contained about 2.6 times more α-tocopherol and plastoquinol and about 1.7 times more total plastoquinone than non-tolerant strains. Exposure to excess copper led to oxidation of the plastoquinone pool in non-tolerant strains, whereas this effect was less pronounced or did not occur in copper-tolerant strains. Peroxidase activity was approximately 1.75 times higher in the tolerant strain than in the non-tolerant one. The increase in peroxidase activity in the tolerant strain was less pronounced when the algae were grown in dim light. In the tolerant line nonphotochemical quenching was induced faster and was usually about 20–30% more efficient than in the non-tolerant line. The improvement of antioxidant defense and photoprotection may be important factors in the evolutionary processes leading to tolerance to heavy metals.

## Introduction

Copper is an essential micronutrient for photosynthetic organisms. The electrochemical potential of Cu^2+^/Cu^+^ is − 268 mV, which is in the physiological range, making this element useful for participating in the catalysis of redox reactions occurring in cells (Nies 1999). Copper is a prosthetic group in many enzymes, including cytochrome oxidase and Cu/Zn superoxide dismutase, and is also found in protein electron carriers, such as plastocyanin. This makes copper essential for the functioning of photosynthesis, respiration, and many other metabolic processes (Nagajyoti et al. 2010). However, copper is highly toxic at supra-optimal concentrations. Photosynthetic organisms are particularly sensitive to excess copper, as the metabolic disruption occurs when the intracellular copper level is only slightly above the optimal level. Since Cu^2+^ is more mobile in the water than in the soil, this heavy metal poses a serious threat to algae and aquatic plants (Fernandes and Henriques 1991). Freshwater ecosystems are particularly threatened by copper contamination resulting from anthropogenic activities, such as mining, smelting, chemical industry, and many others (Nagajyoti et al. 2010; Andresen and Küpper 2013).

The adverse effects of heavy metals on living organisms are pleiotropic (Nagajyoti et al. 2010). Considering copper-induced toxicity, there are two main mechanisms involved. A major target of copper toxicity is the light phase of photosynthesis (Küpper and Andresen 2016). This element is known to interact with Tyr_Z_ and Tyr_D_, nonheme Fe, cyt *b*_*559*_, and sites near the pheophytin, Q_A_ and Q_B_, binding pockets in photosystem II (PS II), leading to inhibition of O_2_ evolution (Burda et al. 2003; Yruela 2005; DalCorso 2012). Copper can also substitute Mg^2+^ in chlorophyll (Chl), leading to a loss of energy excitation (Küpper and Andresen 2016). Apart from its inhibitory effect on light reactions, copper is also known to inhibit enzymes crucial for the dark phase of photosynthesis, such as Rubisco and phosphoenolpyruvate carboxylase, and to damage the chloroplast structure (DalCorso 2012). The second major mechanism of toxicity is related to the redox properties of copper, which allow this element to undergo unwanted and uncontrolled redox cycling in living cells. These reactions lead to the formation of reactive oxygen species (ROS); in particular, the reaction of Cu^+^ with H_2_O_2_ leads to the formation of the most dangerous ROS, the hydroxyl radical. Because of this aspect of copper chemistry, it is included in the group of redox-active heavy metals (DalCorso 2012; Stoiber et al. 2013).

Oxidative stress, which is a situation of excessive ROS production in cells, can be induced by the direct action of copper ions, but also indirectly, as a result of the disturbance of photosynthesis and other metabolic processes (Pinto et al. 2003). Inhibition or slowing of photosynthetic electron transfer leads to overexcitation of the photosynthetic apparatus, resulting in undesirable side reactions that cause damage to pigments and proteins, as well as to the formation of singlet oxygen (^1^O_2_) and superoxide (O_2_^•−^) (Edreva 2005). Therefore, protective mechanisms, such as the thermal dissipation of absorbed light energy, may play a role in alleviating copper toxicity. Thus, nonphotochemical quenching of Chl fluorescence (NPQ) is a mechanism that prevents ROS formation in cells (Finazzi et al. 2006). To cope with ROS already formed, living organisms have evolved robust ROS-detoxifying systems based on low-molecular-weight antioxidants and antioxidant enzymes (Pinto et al. 2003). Since chloroplasts are the major source of ROS in photosynthetic organisms, plastidial antioxidants are very important for antioxidant defense in algae. This group includes hydrophilic compounds, such as ascorbate (Asc) and reduced glutathione (GSH), as well as hydrophobic antioxidants belonging to the prenyllipids. The latter group, which includes carotenoids (Car), tocopherols, and plastoquinol (PQH_2_), is very important for the protection of thylakoid membranes (Nowicka et al. 2021a). Superoxide dismutases (SOD), catalase (CAT), and peroxidases using, e.g., Asc (ascorbate peroxidase, APX), GSH (glutathione peroxidase, GPX), or phenolic compounds as electron donors for peroxides reduction, are examples of ROS-detoxifying enzymes (Gechev et al. 2006). Increased levels of low-molecular-weight antioxidants and antioxidant enzyme activities are considered important for the acquisition of heavy metal tolerance (Nowicka et al. 2016a; Nowicka 2022).

The green microalga *Chlamydomonas reinhardtii* P.A. Dangeard is a model photosynthetic microorganism widely used in the research concerning heavy metal toxicity and tolerance (Hanikenne 2003). This microalga is easy to grow, metabolically profiled, and its genome has been sequenced; all of this makes it useful in experiments (Nowicka et al. 2016a). The short life cycle, the haploid vegetative stage, and the ability to adapt to various stress conditions are characteristics that make *C. reinhardtii* a good model organism for research concerning microevolutionary processes (Hanikenne 2003; Pluciński et al. 2018).

Pollution by heavy metals, such as copper, is a serious environmental problem. Yet, there still exists a paucity of comprehensive understanding of the mechanisms responsible for the formation of tolerance to heavy metals in photosynthetic organisms. While various adaptations have been identified, it is unclear how they arise through microevolutionary processes. Study of heavy metal-tolerant strains of algae can fill this gap. Pluciński et al. (2018, 2021) have obtained copper-tolerant strains of *C. reinhardtii* and aimed to understand the mechanisms responsible for their tolerance to copper. In the current study, we measured various parameters to test for the presence of possible mechanisms involved in algal resistance to stress induced by high concentrations of copper ions in the environment. Specifically, the contents of photosynthetic pigments, prenyllipid antioxidants, i.e., α-tocopherol (α-Toc), PQH_2_, plastoquinone (PQ), peroxidase (POX) activity, and the efficiency of NPQ, were measured in copper-tolerant strains of *C. reinhardtii* and non-tolerant paternal strains exposed to elevated copper concentrations.

## Materials and methods

### Strains used, growth conditions, and determination of photosynthetic pigments

*Chlamydomonas reinhardtii* was cultured aseptically in Erlenmeyer flasks (250 mL) in Sager-Granick (SG) medium supplemented with 100 mM mannitol, 7.5 mM sodium acetate, and 1.7 mM citrate, on a shaker, in a growth chamber at 22 ± 2 °C as described in Pluciński et al. (2018). The non-tolerant parental strain (“N1”) and the non-tolerant cell wall-containing strain 11-32b (“wall”) were grown at a Cu^2+^ concentration of 0.25 μM nominal for SG medium. The copper-tolerant “Cu2” strain was grown for more than a year in modified SG medium with Cu^2+^ concentration increased to 5.25 μM. The copper-tolerant “Cu200” population was obtained from the Cu2 strain as a result of culture in the presence of 200 μM Cu^2+^ for more than a year. Algal cultures of the four experimental populations were inoculated weekly in fresh medium (3 mL of 1-week-old culture per 100 mL of new medium). Algae were cultivated under a 16:8 light:dark cycle with 50 μmol m^−2^ s^−1^ photosynthetically active radiation from a fluorescent lamp.

In the experiment aimed at the evaluation of the photosynthetic pigments and the POX activity, the algae were cultivated on a 48-well plate. The initial amount of chlorophyll (20 ng/mL) was the same for all variants. The N1 and Cu2 strains were grown in either normal (50 μmol m^−2^ s^−1^) or shade (10 μmol m^−2^ s^−1^) light on the media containing either 0.25 or 50 μM Cu^2+^ in six biological replicates.

For measurements of Chl *a*, Chl *b*, and total Car, 200 µL of cell suspensions were centrifuged (5 min, 9000 g). The resulting pellet was extracted with acetone, the extract was centrifuged again (5 min, 9000 g) to remove cell debris, and the photosynthetic pigment content was determined spectrophotometrically according to Lichtenthaler (1987).

In the experiment aimed at evaluating prenyllipid antioxidants, N1, wall, Cu2, and Cu200 strains were grown in Erlenmeyer flasks (250 mL) in control medium containing 0.25 μM Cu^2+^ and in media containing 25, 50, 100, and 200 μM Cu^2+^ in four replicates. The initial amount of Chl was the same in all variants (20 ng/mL). Samples (10 mL per each) were collected after 7 days of culture growth and centrifuged (5 min, 5400 g, 4 °C). The pellet obtained was frozen in liquid nitrogen and then extracted as described in the “Determination of prenyllipids” section.

In the experiment aimed at evaluating of the Chl fluorescence parameters, the N1 and Cu2 strains were grown under normal light (50 μmol m^−2^ s^−1^) in the media containing either 0.25 or 50 μM Cu^2+^ in 8 replicates, in a 48-well plate. The initial amount of Chl was the same in all variants (20 ng/mL). Four pre-incubation variants were used: 2 h under 50 μmol m^−2^ s^−1^ light or 2 h in darkness; in the presence or absence of a solution containing an organic carbon and phosphorus source. The solution used contained sodium acetate, sodium citrate, K_2_HPO_4_, and KH_2_PO_4_. The addition of the solution (20 µL for 1 mL of culture) resulted in 80% increase in the concentrations of the listed components in the medium. After preincubation, the plates were dark adapted for 20 min and the Chl fluorescence parameters were measured.

### Determination of peroxidase activity

Peroxidase activity was determined by a classical colorimetric assay using pyrogallol (Maehly and Chance 1954). Pyrogallol oxidation by peroxidases using ascorbate and polyphenolic compounds as electron sources in vivo has been described previously (Van Doorn and Ketsa 2014; Shigeoka et al. 2002). Samples (2 mL of cultures) were centrifuged (3 min, 12000 g), resuspended in 250 µL of 100 mM phosphate buffer pH 6.0, and then freeze-thawed/thawed three times in liquid nitrogen to disrupt the cells. The resulting suspension was centrifuged again (5 min, 12000 g) to remove cell debris. The following procedure was used: 200 µL of extract was added to the reaction mixture (500 µL of distilled water + 100 µL of 100 mM phosphate buffer pH 6.0 + 100 µL of 0.5% pyrogallol solution in 100 mM phosphate buffer pH 6.0), then 100 µL of 3% H_2_O_2_ was added. The whole mixture was mixed by pipetting and the increase in purpurogallin concentration was monitored by absorbance detection at *λ* = 420 nm at 20 °C. POX activity was expressed as the change in absorbance per second, normalized to the Chl content, and multiplied by 1000.

### Determination of prenyllipids

Extraction of prenyllipids and determination of α-Toc, PQH_2_, and PQ by RP-HPLC were performed as described in Nowicka and Kruk (2012). To avoid oxidation of PQH_2_ during acetone extraction, the sample was incubated with the solvent for no longer than 2 min, then centrifuged (5 min, 9000 g), evaporated in a stream of nitrogen, dissolved in 200 µL of methanol, and injected into an HPLC system. The HPLC analysis of the prenyllipids was carried out in the following system: C_18_ reverse-phase column (Teknokroma, Spain, 5 µm, 25 cm × 0.4 cm), eluent methanol:hexane (340:20, v/v), flow rate of 1.5 mL/min, absorbance detection at *λ* = 255 nm, fluorescence detection at *λ*_ex_ = 290 nm, *λ*_em_ = 330 nm. The concentration of prenyllipids in the extracts was evaluated by comparison with the respective standards and normalized on the basis of the total Chl content (mol/100 mol Chl *a* + *b*).

### Chlorophyll fluorescence parameters

Chl fluorescence parameters were measured using an Open FluorCam FC 800-O (Photon Systems Instruments, Brno, Czech Republic) as described in Nowicka et al. (2016a). Weak red modulated light was used as the measuring light, and white light with an intensity of 2600 μmol photons m^−2^ s^−1^ was used as the saturating light (pulse duration 1000 ms). Red light with an intensity of 220 μmol photons m^−2^ s^−1^ was used as the actinic light. The measured parameter was NPQ, calculated as (*F*_m_ − *F*_m′_)/*F*_m′_ (*F*_m_, maximum fluorescence; *F*_m’_, maximum fluorescence in the sample exposed to actinic light) (Maxwell and Johnson 2000). The induction of NPQ was measured during 30 min of illumination with actinic light, and then the relaxation of NPQ was monitored in the dark for 25 min. Saturating pulses were applied as shown in Fig. 3.

### Statistical analyses

Factors affecting POX activity, Chl *a*, Chl *b*, and Car concentrations were first analyzed by full-factorial linear models with line (N1 and Cu2), copper exposure and light exposure, and all their interactions as fixed factors. The models were then reduced by stepwise removal of non-significant interactions. In the reduced models, the effect of light was always significant. Finally, the analyses were performed separately for each of the two light conditions.

Factors affecting the concentrations of α-Toc, PQH_2_ and PQ, total PQ (PQ + PQH_2_), and the ratio of PQ to total PQ were first analyzed using linear models with line (wall, N1, Cu2, and Cu200) introduced as categorical factor and copper concentration as linear and quadratic predictor. To further explore the interaction of interest, i.e., the interaction between line and copper concentration, the analyses were performed separately for each line.

NPQ induction values in Cu2 and N1 lines were first plotted against incubation time separately for cultures without and with carbon, without and with copper exposure, and in light and dark pre-treatments. The highest value on each curve was then analyzed separately for the two carbon conditions. Both analyses were full-factorial linear models with line (N1 and Cu2), copper exposure and light pretreatment, and all their interactions introduced as fixed factors.

Analyses were conducted in R-4.0.5 (R Core Team 2022), using the lm() function, which is generic to R. Tables of ANOVA results were generated using the Anova() function with the type III sum of squares from the *car* package (Fox and Weisberg 2019). Figures were prepared in the *ggplot2* package (Wickham 2016).

### Data availability

The datasets and R scripts used are stored in the Open Science Framework repository (https://osf.io/gyq42/).

## Results

### Photosynthetic pigments

**Fig. 1:**
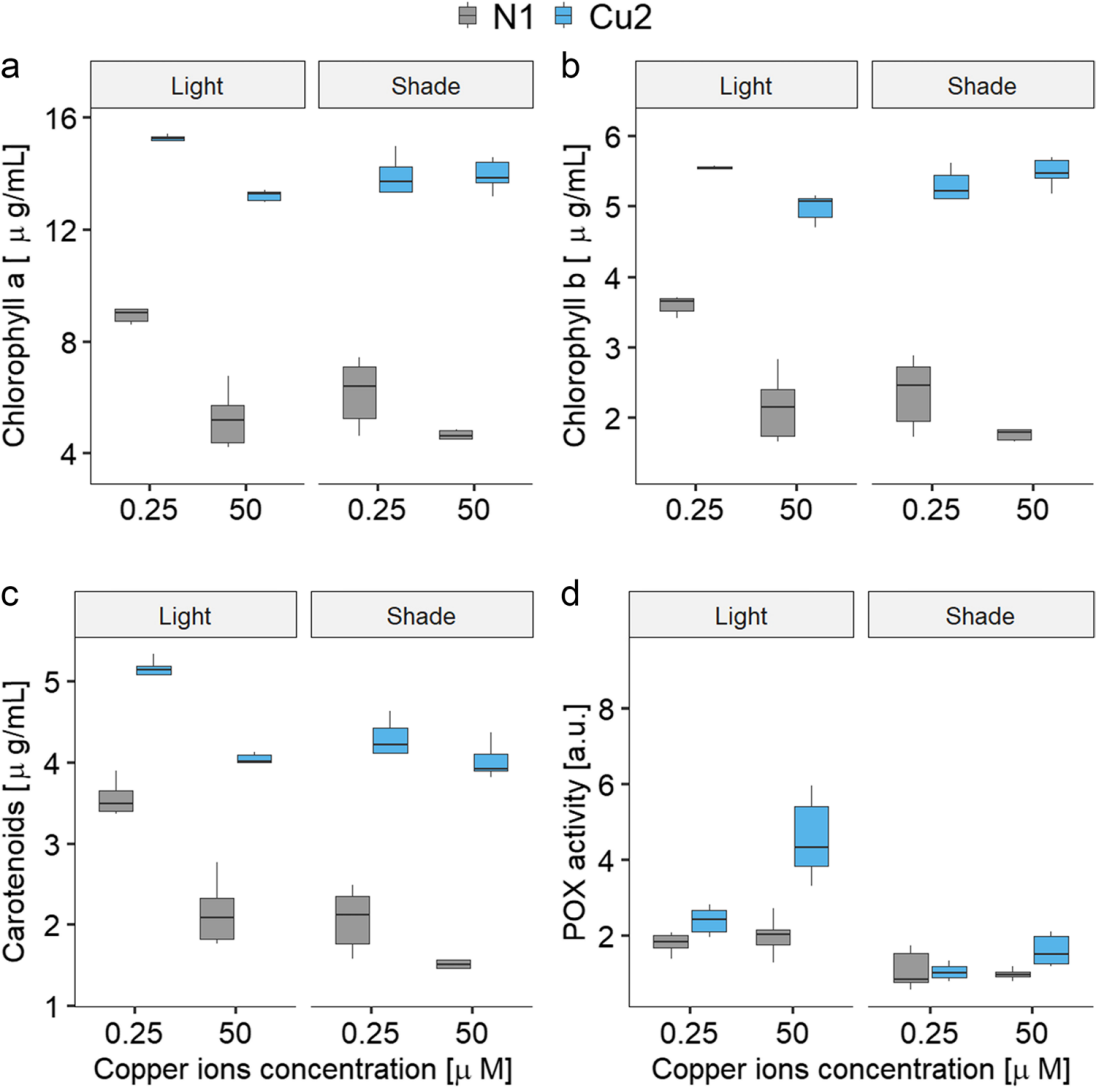
Photosynthetic pigment content and peroxidase (POX) activity in the two experimental lines (N1 and Cu2) in relation to copper exposure in light and shade conditions: **a** chlorophyll *a*, **b** chlorophyll *b*, **c** total carotenoid concentrations, and **d** peroxidase activity. The median values are presented with quartiles. The results of the relevant statistical analysis are presented in Tables 1 and 2

The levels of Chl *a*, Chl *b*, and total Car were higher in the copper-tolerant Cu2 line cultures than in the non-tolerant N1 line cultures (Fig. 1). The presence or absence of elevated copper and the two light conditions influenced the magnitude of these differences.

Chl *a* concentration was shaped by all the main factors in the model and also by the interactions of line × copper exposure, line × light, and copper × light (Table 1). Analyses carried out separately for light and shade conditions revealed that the effects of copper exposure were stronger in light than in shade (Table 2). In all but one combination of factors, exposure to copper reduced the Chl *a* content compared to treatment without copper. The exception was the Cu2 line measured in the shade, where the Chl *a* concentration was not affected by the addition of copper (Fig. 1a). In the N1 strain, exposure to higher light led to an increase in Chl *a* content both in the presence and absence of excess copper. However, this effect was less pronounced in copper-exposed algae. For the Cu2 strain, a slight increase in Chl *a* was observed for the control culture exposed to higher light, compared to the series grown in the shade, while the opposite trend was observed for the copper-exposed Cu2 (Fig. 1a).

**Table 1:** Results of the linear model in which the activity of peroxidase (POX), chlorophyll *a* (Chl *a*), chlorophyll *b* (Chl *b*), and carotenoids (Car) concentrations were analyzed with respect to the experimental line, copper exposure, and light exposure, and significant interactions of those factors

		POX activity			Chl *a*		Chl *b*		Car	
	*df*	*F*	*p*	*df*	*F*	*p*	*F*	*p*	*F*	*p*
Line	1	9.6	0.003	1	352.8	< 0.001	327.3	< 0.001	194.9	< 0.001
Copper	1	6.1	0.017	1	12.9	< 0.001	9. 1	0.0044	27.4	< 0.001
Light	1	8.9	0.004	1	4.7	0.035	4.5	0.039	26.5	< 0.001
Line × copper	1	7.9	0.007	1	11.0	0.002	16.8	< 0.001	4.1	0.049
Line × light				1	5.4	0.024	13.1	< 0.001	7.1	0.011
Copper × light				1	7.6	0.009	7.31	0.009	8.8	0.005
Residuals	43			41						

*Df*, degrees of freedom; *F*, calculated statistics; *p*, probability of obtaining a result by chance

**Table 2:** Results of the linear model in which peroxidase (POX) activity, chlorophyll *a* (Chl *a*), chlorophyll *b* (Chl *b*), and carotenoids (Car) concentrations were analyzed with respect to the exposure of the experimental line and copper and the interactions of those factors under (a) light conditions and (b) in shade

		POX activity		Chl *a*		Chl *b*		Car	
	*df*	*F*	*p*	*F*	*p*	*F*	*p*	*F*	*p*
*Light*									
Line	1	38.2	< 0.001	117.0	< 0.001	89.3	< 0.001	48.1	< 0.001
Copper	1	21.2	< 0.001	17.4	< 0.001	14.0	0.001	25.6	< 0.001
Line × copper	1	14.8	0.001	5.2	0.033	8.3	0.009	1.9	0.185
Error	20								
*Shade*									
Line	1	1.0	0.318	249.9	< 0.001	270.2	< 0.001	187.5	< 0.001
Copper	1	0.9	0.361	0.4	0.541	0.0	0.823	3.5	0.076
Line × copper	1	2.4	0.134	5.6	0.028	8.2	0.009	2.3	0.146
Error	20								

The Chl *b* concentration showed a similar pattern to that of Chl *a* (Fig. 1, Table 1). In particular, it occurred in terms of the weaker effects of the line and the exposure to copper in the shade compared to the light conditions (Table 2) and the absence of decline of Chl *b* in the Cu2 line exposed to copper and measured in shade (Fig. 1b).

The concentration of Car was shaped by all the main factors in the model and also by the interactions of line × copper exposure, line × light, and copper × light (Table 1). It was clearly higher in Cu2 compared to the N1 line and lower in samples exposed to copper. Analyses carried out separately for light and shade conditions showed that in each of these conditions, the effects of line and the effect of copper exposure were visible, but these two factors did not interact (Table 2, Fig. 1c). The response of Car to light conditions was similar to that observed for Chls (Fig. 1c).

### Peroxidase activity

POX activity was affected by all the main factors in the model and by the interaction between line and copper exposure (Table 1, Fig. 1d). In short, it was on average higher in Cu2 compared to the N1 line, in samples exposed to 50 µM Cu^2+^ compared to the control, and in light conditions compared to shade. Analyses carried out separately for light and shade showed that the effects of line and copper were pronounced only under light conditions. The highest value of POX activity was found in the Cu2 line exposed to 50 µM Cu^2+^ (Table 2a). In the shade, none of the factors differentiated the peroxidase activity (Table 2b).

### Prenyllipid content

**Fig. 2:**
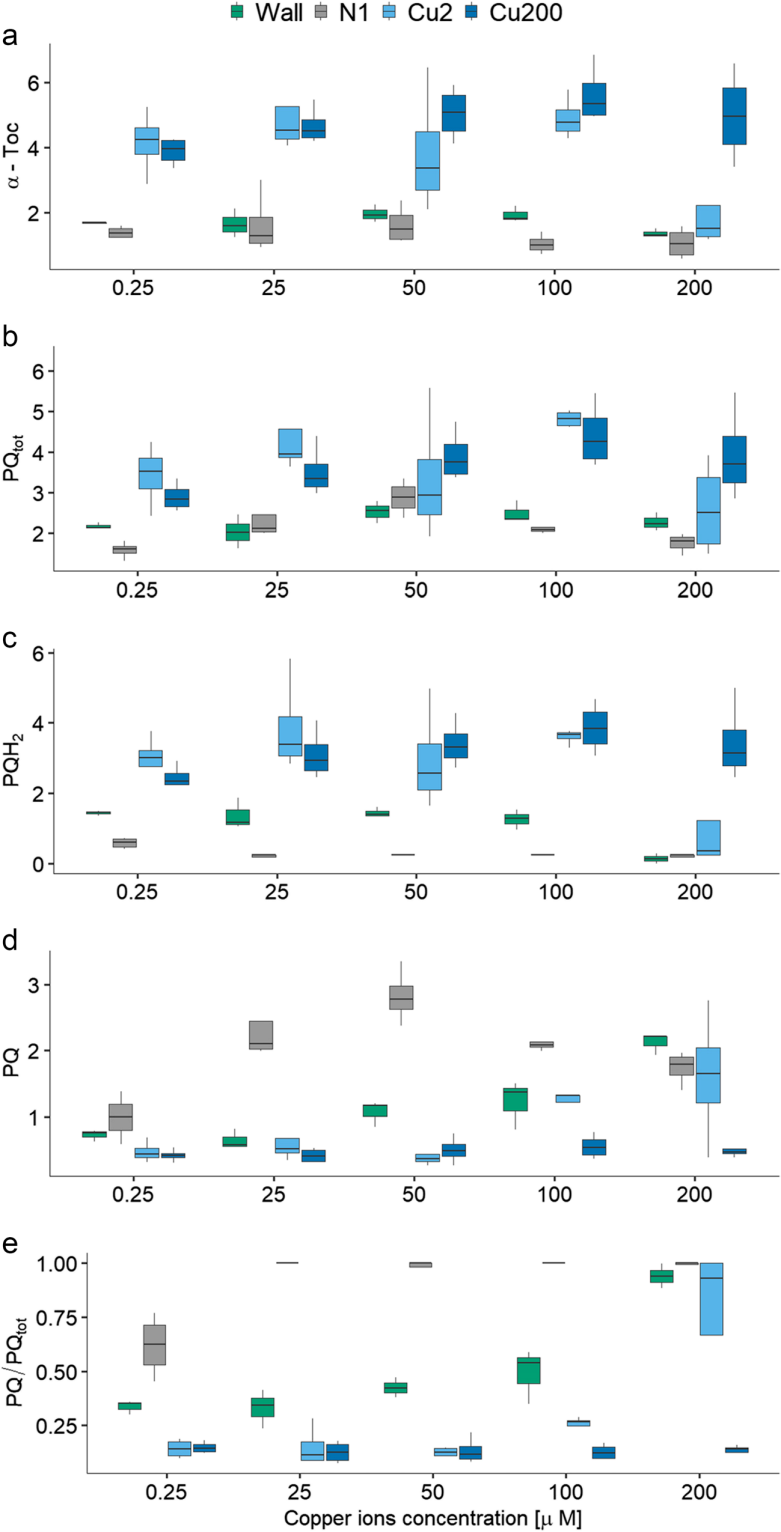
Prenyllipid content in the four experimental lines (wall, N1, Cu2, and Cu200) in relation to a range of copper concentrations. **a** α-Toc, **b** total PQ (PQ_tot_), **c** PQH_2_, **d** PQ, and **e** PQ/PQ_tot_. The median values are presented with quartiles. The prenyllipid content was normalized to the total Chl content and expressed in [mol/100 mol Chl *a* + *b*]. The results of the relevant statistical analysis are presented in Tables 3 and 4

The concentration of α-Toc was characterized by the significant interaction between the line and the copper concentration (Table 3, Fig. 2a). The interaction stemmed from the fact that the four lines responded differently to copper. Specifically, the copper-tolerant Cu200 line, which had the highest level of α-Toc on average, showed a quadratic response to increasing copper concentration and had the highest α-Toc level for 100 µM Cu^2+^. The Cu2 line showed increasing α-Toc levels at lower copper concentration and a marked decrease at 200 µM Cu^2+^, but the quadratic relationship with copper concentration was only marginally significant (Table 4). The non-tolerant lines N1 and wall showed generally low α-Toc concentrations. The wall line showed a quadratic effect of copper concentration, but this was much less pronounced than the other two lines. Similar to Cu2, there was a decrease in α-Toc content for the highest copper concentration applied (Fig. 2a). Line N1 was insensitive to copper concentration (Table 4).

**Table 3:** Results of linear model in which α-tocopherol (α-Toc), plastoquinol (PQH_2_) and plastoquinone (PQ), total PQ (PQ_tot_ = PQH_2_ + PQ), and PQ/PQ_tot_ ratio were analyzed by linear models with line, copper concentration introduced as linear and quadratic term, and their interaction

		α-Toc		PQH_2_		PQ		PQ_tot_		PQ/PQ_tot_	
	*df*	*F*	*p*	*F*	*p*	*F*	*p*	*F*	*p*	*F*	*p*
Line	3	34.9	< 0.001	36.0	< 0.001	25.5	< 0.001	12.6	< 0.001	62.4	< 0.001
Copper	1	5.7	0.019	3.2	0.080	11.4	0.001	14.5	< 0.001	2.8	0.100
Copper^2^	1	9.1	0.003	7.1	0.009	4.9	0.031	16.0	< 0.001	0.2	0.624
Line × copper	3	5.7	0.002	6.9	< 0.001	6.1	0.001	2.6	0.063	12.0	< 0.001
Error	66

**Table 4:** Results of regression analyses of α-tocopherol (α-Toc), plastoquinol (PQH_2_) and plastoquinone (PQ), total PQ (PQ_tot_ = PQH_2_ + PQ), and the PQ/PQ_tot_ ratio with respect to copper concentration, separately for each line. The quadratic term was removed from the model if its *p* > 0.1

Copper				Copper^2^		
	*F*	*df*	*p*	*F*	*df*	*p*
*α-Toc*						
Wall	3.06	1, 12	0.106	6.84	1, 12	0.023
N1	2.47	1, 18	0.134			
Cu2	7.86	1, 17	0.0122	3.62	1, 17	0.0741
Cu200	2.9	1, 17	0.105	6.52	1, 17	0.020
*PQH*_*2*_						
Wall	58.8	1, 12	< 0.0001	9.4	1, 12	0.009
N1	11.5	1, 17	0.003	13.7	1, 17	0.002
Cu2	8.25	1, 17	0.012	3.80	1, 17	0.068
Cu200	3.33	1, 17	0.086	5.27	1, 17	0.035
*PQ*						
Wall	81.8	1, 13	< 0.0001			
N1	0.02	1, 17	0.890	11.3	1, 17	0.004
Cu2	17.2	1, 18	0.0006			
Cu200	0.9	1, 18	0.345			
*PQ*_*tot*_						
Wall	0.44	1, 13	0.518			
N1	0.92	1, 17	0.351	7.56	1, 17	0.014
Cu2	1.40	1, 18	0.253			
Cu200	4.01	1, 17	0.061	6.33	1, 17	0.022
*PQ/PQ*_tot_						
Wall	130	1, 12	< 0.0001	6.99	1, 12	0.021
N1	9.88	1, 17	0.006	13.1	1, 17	0.002
Cu2	24.1	1, 18	0.0001			
Cu200	0.097	1, 18	0.760			

The total PQ content showed the highest values for the Cu2 and Cu200 lines, while it was lower for the wall and N1 lines (Fig. 2b). A quadratic relationship with copper concentration was observed for all lines (Fig. 2b), but was significant for the Cu200 and N1 lines (Table 4). The total PQ content did not change significantly when the wall line was exposed to excess copper. The N1 and Cu2 strains showed an increase in total PQ at lower copper concentrations applied and then a decrease. For the N1 strain, this decrease was observed at 100 and 200 µM Cu^2+^, whereas for Cu2 strain only for 200 µM Cu^2+^. An increase in the total PQ level was observed in the Cu200 strain exposed to copper for all copper concentrations tested compared to the control series.

The PQH_2_ level showed a similar pattern to that of α-Toc (Table 3, Fig. 2c). In the Cu200 and Cu2 lines, the PQH_2_ levels were high. The PQH_2_ content in these lines was affected by the copper concentration in a nonlinear manner, with the PQH_2_ levels initially increasing with the increase in the copper concentration and decreasing for the 200 µM Cu^2+^. In the case of the Cu2 line, this decrease was very pronounced, almost reaching the levels observed for 200 µM Cu^2+^ in the other two lines, although the quadratic term showed only marginal significance (Table 4). The wall line showed a quadratic response to copper concentration, and the N1 line had hardly any PQH_2_ except at 0.25 µM Cu^2+^. Exposure to elevated copper caused the oxidation of almost all PQH_2_ in the N1 strain at all the Cu concentrations applied and in the wall and Cu2 strains at the highest copper concentration applied.

The PQ content showed the highest values for the N1 line, but with a quadratic relationship with the copper concentration (Fig. 2d). In the case of the wall line, PQ had intermediate levels, and it actually increased with increasing copper concentration. The Cu2 line had low levels of PQ at low copper concentration, but these levels increased at 100 and 200 µM Cu^2+^. No such increase was observed in the Cu200 line, which maintained the lowest levels of PQ and was not affected by the copper concentration (Table 4, Fig. 2d).

The ratio of PQ to total PQ differed greatly between the lines, as did the response of lines to copper concentrations, as indicated by the significant line-copper interaction (Table 3, Fig. 2e). For N1 strain PQ/PQ_tot_ reached the values of 1 for all copper concentrations except the 0.25 µM Cu^2+^. The proportion of PQ in the total plastoquinone pool (PQH_2_ + PQ) of the wall line increased exponentially, starting from intermediate values. Increasing copper concentration caused PQ/PQ_tot_ to increase exponentially in the Cu2 line, but not in the Cu200 line.

### Photosynthetic parameters

**Fig. 3:**
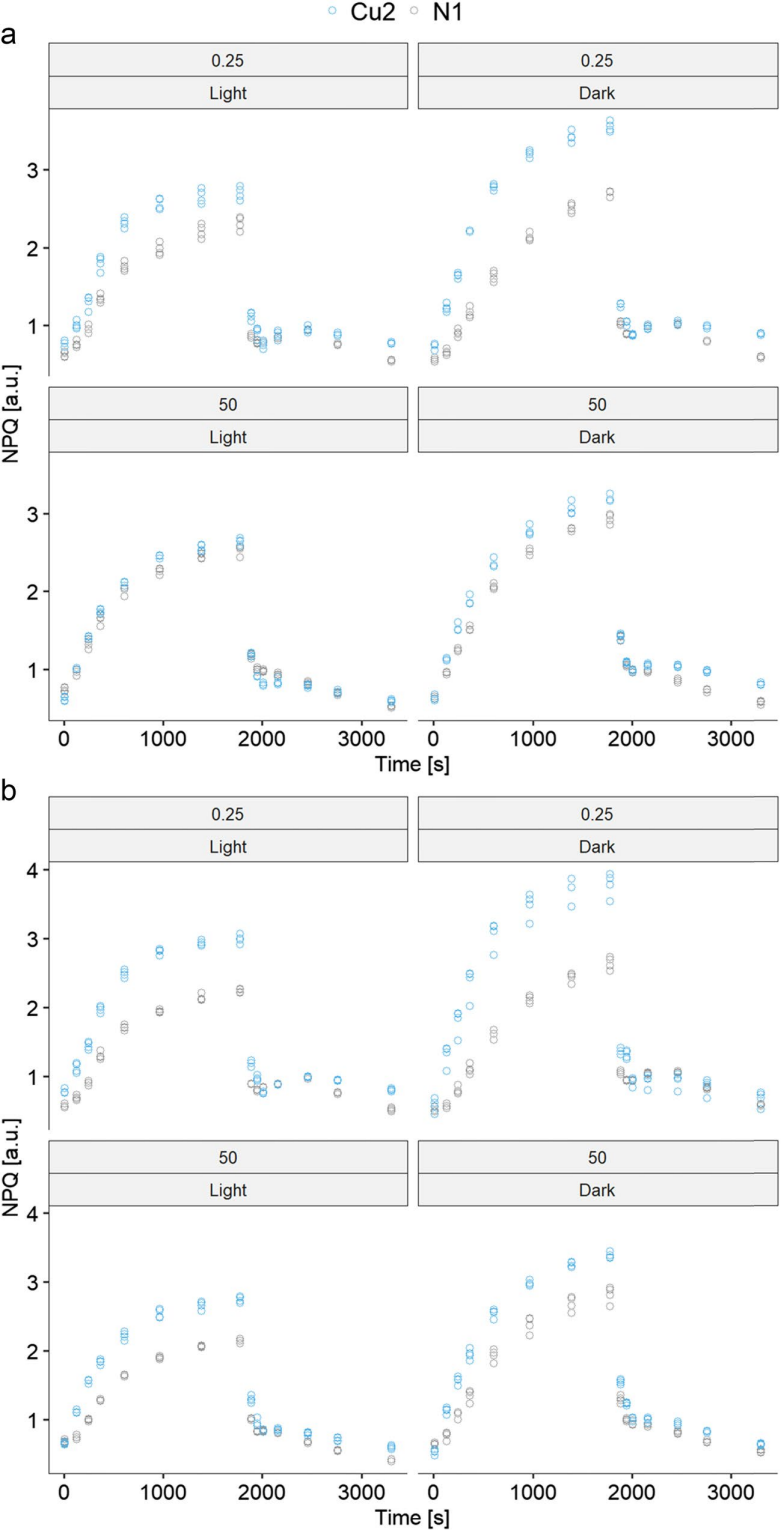
Nonphotochemical quenching (**NPQ**) of chlorophyll fluorescence over time in experimental lines (N1 and Cu2) in **a** absence and **b** presence of additional nutrients (citrate, acetate, and phosphate ions). The numbers above the subgraphs (0.25 and 50) indicate the µM concentration of copper ions in the variant of the experiment whose results are presented. The curves were plotted for all combinations of light or dark pretreatments and absence or presence of copper exposure. The results of the corresponding statistical analysis are presented in Table 5

The copper-adapted line, Cu2, showed increased efficiency of NPQ induction compared to the N1 line, in both copper-exposed and control cultures, irrespective of light pre-treatment and carbon exposure (Fig. 3). The induction of NPQ in the Cu2 line was usually faster than in the N1 line (Fig. 3). Statistical analyses of NPQ maxima showed that all factors and their interactions were influential (Table 5). Specifically, NPQ induced in dark pretreated cultures was more efficient than NPQ induced in light pretreated cultures. In the Cu2 line, the effect of darkness was more pronounced than in N1. This trend was observed in both control and copper-exposed algae. Exposure to copper led to a decrease in NPQ in Cu2, while copper-induced stress generally led to a slight increase in NPQ efficiency in the N1 line (Table 5, Fig. 3). Additional nutrients (citrate, acetate, and phosphate ions) increased the differences in NPQ between the measured strains (Fig. 3).

**Table 5:** Results of the linear model in which the maximum value of the NPQ efficiency was analyzed with respect to line, copper exposure, and light or dark pretreatment and their interactions. Analyses were run separately for (a) cultures without an additional amount of organic carbon and (b) cultures with the addition of organic carbon

	(a) Without carbon			(b) With carbon		
	*F*	*df*	*p*	*F*	*df*	*p*
Line	341.87	1, 24	< 0.0001	584.29	1, 24	< 0.0001
Light	606.36	1, 24	< 0.0001	392.00	1, 24	< 0.0001
Copper	0.43	1, 24	0.520	18.44	1, 24	0.0002
Line × light	52.45	1, 24	1.75e − 07	8.65	1, 24	0.0071
Line × copper	100.52	1, 24	< 0.0001	33.69	1, 24	< 0.0001
Light × copper	8.89	1, 24	0.006	0.91	1, 24	0.349
Line × light × copper	14.27	1, 24	< 0.0001	10.63	1, 24	0.0033

## Discussion

### Photosynthetic pigments

The results of photosynthetic pigments measurements show that light increases the toxicity of copper. In particular, this can be inferred from the observed decrease in the pigment levels in Cu2 exposed to copper grown in normal light, when compared to the corresponding series grown in shade (Fig. 1). This finding is consistent with previous reports (Lu and Zhang 1999; Knauert and Knauer 2008; Nielsen and Nielsen 2010).

### Role of peroxidase

The importance of antioxidant enzymes for acclimation to heavy metal-induced stress has been widely documented (Mourato et al. 2012; Sytar et al. 2013; Nowicka 2022). Increased activities of SOD, CAT, and APX were observed in copper-exposed *C. reinhardtii* (Zheng et al. 2011; Jiang et al. 2016; Nowicka et al. 2021b). Peroxidase activity was increased in the copper-exposed green algae *C. reinhardtii*, *Scenedesmus acuminatus*, *Chlorella vulgaris*, and in the diatom *Odontella mobiliensis* (Manimaran et al. 2012; El-Naggar and Sheikh 2014; Jiang et al. 2016; Hamed et al. 2017).

The observed increase in POX activity in copper-exposed N1 and Cu2 strains (Fig. 1d) confirms the role of peroxidases in response to copper-induced stress. The fact that this effect is more pronounced in algae grown at higher light intensity supports the hypothesis that light enhances copper toxicity. The effect of light conditions on POX activity suggests that peroxidases play a role in protecting *C. reinhardtii* from ROS generated during photosynthesis. The increased activity of POX in the Cu2 line compared to N1 may suggest that the increase in POX activity plays a role in long-term acclimation.

### Role of the prenyllipids

Acclimation to heavy metal-induced stress is often accompanied by an increase in the antioxidant levels in the exposed organism. The extent of this increase is usually proportional to the concentration of the heavy metal salt applied. The application of concentrations high enough to cause severe stress results in the depletion of antioxidants (Elbaz et al. 2010; Nowicka et al. 2016b, 2020, 2021b).

The increase in α-Toc levels in response to copper has been observed in both higher plants and algae (Zengin and Munzuroglu 2005; Luis et al. 2006; Collin et al. 2008; Nowicka et al. 2016a; Hamed et al. 2017). Experiments on *C. reinhardtii* showed that the increase in α-Toc content usually occurs at lower copper concentrations applied, whereas at higher concentrations the α-Toc level decreases, most probably due to enhanced oxidative degradation of this compound (Luis et al. 2006; Nowicka et al. 2016a). An increase in the content of PQH_2_ and the total PQ pool was observed in *C. reinhardtii* exposed to chromium and cadmium salts (Nowicka et al. 2016a, 2020). On the other hand, *C. reinhardtii* 11-30b strain (referred to the wall strain in this article) exposed to copper concentrations high enough to significantly inhibit Chl synthesis showed an increased PQ/PQ_tot_ ratio. This effect probably resulted from the enhanced oxidation of PQH_2_ due to ROS scavenging or inhibition of PQ reduction in PS II, or both of these mechanisms (Nowicka et al. 2016a).

The increased α-Toc, PQH_2_, and PQ_tot_ levels observed in copper-adapted strains (Fig. 2, Table 3) suggest that the accumulation of these prenyllipid antioxidants may be an important mechanism to confer greater tolerance to copper. It is also worth noting that when the Cu200 strain, adapted to the medium containing 200 µM Cu^2+^, was grown at lower copper concentrations, the prenyllipid content decreased. This effect could be the result of a reduced demand for these compounds.

The decrease in α-Toc and PQ_tot_, as well as the increased proportion of PQ in the total PQ pool in the wall, N1, and Cu2 strains exposed to copper (Fig. 2, Table 4), is an indicator of the occurrence of increased copper-induced oxidative stress. The Cu2 strain shows such an effect in the presence of 200 µM Cu^2+^, despite having “basal” α-Toc and PQ_tot_ levels similar to Cu200. This observation suggests that the increased content of these compounds is not the only mechanism responsible for the improvement in copper tolerance. The fact that the decrease in prenyllipids and the increased oxidation of PQH_2_ in the N1 strain occur in the presence of Cu^2+^ concentrations, for which there is no such effect in cell wall-containing strain, supports the hypothesis that the complexation of heavy metal ions by the cell wall is an important protective mechanism (Fig. 2) (Macfie et al. 1994; Prasad et al. 1998; Macfie and Welbourn 2000).

### Role of NPQ

A copper dose-dependent increase in NPQ efficiency has been observed in *C. reinhardtii* after 2 weeks of exposure (Nowicka et al. 2016a). The increase in the qN parameter, which also refers to the non-photochemical quenching capacity of Chl fluorescence, was also observed in *C. reinhardtii* after 96 h of copper exposure (Juneau et al. 2002). The much faster and more pronounced induction of NPQ observed in the Cu2 strain (Fig. 3) suggests that NPQ plays a role in the adaptation to elevated copper concentrations. Since the observed difference concerns the rapidly relaxing NPQ component, the enhanced NPQ is the most likely the result of a more efficient qE. In *C. reinhardtii*, this mechanism is strongly dependent on the level of the protective protein LHCSR3 (Bonente et al. 2011). Interestingly, unlike in higher plants, the xanthophyll cycle pigments are less important for qE in *C. reinhardtii*. However, their content increases in algae acclimated to grow at higher light intensities (Quaas et al. 2015). The increase in NPQ observed in dark-preincubated algae compared to light-preincubated algae (Fig. 3) may be due to the fact that light-preincubated *C. reinhardtii* has its metabolism “tuned” to photosynthesis, allowing rapid consumption of light phase products. This would result in a lower pH gradient across thylakoid membranes compared to actinic light-exposed dark-preincubated algae. Due to the regulatory role of pH gradient in qE, the lower pH gradient results in the lower efficiency of qE (Bonente et al. 2011).

The presence of organic carbon has a positive effect on algal growth despite a down-regulation of photosynthetic efficiency under mixotrophic growth conditions (Johnson and Alric 2012). An additional energy source modulates fatty acid metabolism and glycolysis and increases the proportion of cyclic electron transport in the reactions of the light phase of photosynthesis (Chapman et al. 2015). The addition of acetate to the medium induces an increase in the NPQ due to the ∆pH-dependent down-regulation of PS II and the transition from state 1 to state 2 (Endo and Asada 1996). The first of the aforementioned mechanisms is similar to that which occurs during high light exposure (Tian et al. 2019). The increased activity of cyclic electron transport is associated with the transition from states 1 to 2 (Finazzi 2005) and has been shown to be a form of protection of PS II from the negative effects of Cd^2+^ (Wang et al. 2013). Algae grown in the presence of acetate are less susceptible to photoinhibition (Roach et al. 2013).

In N1 line, preincubation in light and in the presence of acetate had a negative effect on the increase in maximum NPQ in algae exposed to copper (Fig. 3, Table 5). This may be due to the protective role of organic carbon present in the medium. The physiological mechanism underlying the effect of preincubation conditions on maximum NPQ will be the subject of our future research.

## Conclusions

Wall-less *Chlamydomonas reinhardtii* strains adapted to elevated copper levels exhibit several mechanisms that protect them from excessive concentrations of this heavy metal. Our study shows that these mechanisms include a protective role of peroxidases, increased levels of α-tocopherol and plastoquinol, and increased efficiency of non-photochemical quenching of chlorophyll fluorescence. The enhanced non-photochemical quenching helps to prevent the formation of reactive oxygen species in the photosynthetic apparatus, while the increase in antioxidant content helps to detoxify them. The efficiency of these mechanisms is further modulated by light exposure, which increases copper toxicity. This can be seen as a more pronounced decrease in photosynthetic pigment content and an increase in peroxidase activity in algae exposed to excess copper in normal versus dim light. Based on analyses of prenyllipid content, we suggest that changes in the ratio of reduced to oxidized plastoquinone are the best indicator of the stress response induced by redox-active heavy metals such as copper. Our findings emphasize the role of the analyzed protective mechanisms in the evolution of copper tolerance. Copper tolerant strains of microalgae have potential applications in wastewater treatment.

## Data Availability

The data sets and the R scripts used are stored in the Open Science Framework repository (https://osf.io/gyq42/).

## Abbreviations

APX: Ascorbate peroxidase
Asc: Ascorbate
Car: Carotenoids
Chl: Chlorophyll
df: Degrees of freedom
F: Calculated statistics
GSH: Reduced glutathione
NPQ: Nonphotochemical quenching of chlorophyll fluorescence
*p*: Probability of obtaining a result by chance
POX: Peroxidase
PQ: Plastoquinone
PQH_2_: Plastoquinol
PQ_tot_: Sum of plastoquinol and plastoquinone
PS II: Photosystem II
ROS: Reactive oxygen species
α-Toc: α-Tocopherol
